# Review and case report of the treatment in a young girl with primary failure of eruption

**DOI:** 10.1002/ccr3.5632

**Published:** 2022-03-17

**Authors:** Mirna G. Awad, Lana Dalbah, M. Srirengalakshmi, Adith Venugopal, Nikhilesh R. Vaid

**Affiliations:** ^1^ European University College DHCC Dubai UAE; ^2^ Department of Orthodontics Saveetha Dental College Saveetha Institute of Medical and Technical Sciences Chennai India; ^3^ Department of Orthodontics University of Puthisastra Phnom Penh Cambodia

**Keywords:** ankylosed teeth, infraocclusion, mechanical failure of eruption, non‐syndromic eruption failure, PFE, posterior open bite, primary failure of eruption, *PTH1R* mutation, submerged teeth

## Abstract

Any localized non‐eruption of teeth can be attributed to myriad of factors. A failure of a permanent tooth to erupt or cessation of initial eruption with no obvious local/systemic causative factor is said to be primary failure of eruption (PFE). The etio‐pathogenesis of PFE is due to the mutation of *PTH1R* gene. Clinical features such as infra‐occluded teeth, posterior open bite, lack of any cause or habit are usually attributed to diagnosing the condition, and a confirmatory diagnosis is done by the gene analysis of *PTH1R* gene. Treatment of such a condition is tricky as any application of orthodontic traction to teeth affected by PFE will not be successful and may cause ankylosis. This correspondence reviews and demonstrates the treatment of a case of PFE to restore function and esthetics to the best possible outcome.

## Graphical abstract

Primary failure of eruption is the failure of a permanent tooth to erupt due to no clear local or systemic cause. Orthodontic therapy is challenging and any attempt to move teeth will result in ankylosis, necessitating a multidisciplinary approach.

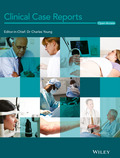

## 1 INTRODUCTION

Tooth eruption is a localized event that is genetically predetermined and occurs at a chronologically predetermined time. Eruption of permanent teeth is usually expedited by resorption of the overlying bone, primary tooth root, and alveolar mucosa.^1^, ^2^

A variety of reasons might contribute to a tooth's failure to erupt into the oral cavity. Mechanical obstructions such as other teeth, calcifications in the surrounding bone,^3^ a few habits such as thumb or digit sucking, conditions in which the cementum fuses to the adjacent bone resulting in ankyloses, which prevents further eruption of the affected tooth are some of the most commonly encountered factors. When an impediment is the cause, eruption generally continues once the obstruction is removed.^1^, ^3^

Primary failure of eruption (PFE) was originally described by Proffit and Vig^4^ and is characterized by a non‐syndromic eruption failure of permanent teeth in the absence of any mechanical obstruction. The condition is found to be inherited and is attributed to the mutation in *PTH1R* gene and the genes involved in activation of cAMP/PKA pathway in tooth eruption.^5^

### 1.1 Features of primary failure of eruption

The characteristic features of this condition are as follows^6^, ^7^, ^8^, ^9^, ^10^, ^11^, ^12^

- History—Familial involvement is one of the signs, but it is not always present and sometimes it follows an incomplete penetrance pattern. However, mutation in parathyroid hormone receptor 1 (*PTH1R*) has been strongly associated.
- Clinical sign—Two clinical parameters that will guide diagnosis of PFE are as follows: involvement of the first permanent molar and supracrestal presentation of affected teeth, in which the eruption pathway is completely clear of obstruction and clear of alveolar bone occlusal to the tooth. Other hallmark clinical features that, if present, can help support a diagnosis of PFE are involvement of the second premolar and the second molar, multiple adjacent teeth affected, bilateral presentation, involvement of teeth in both arches, Class III malocclusion, and concurrent dental anomalies.
- Infraocclusion of the affected teeth often leads to posterior open bite accompanying normal vertical facial growth and inability to move affected teeth orthodontically.
- Site of involvement—Posterior teeth are more frequently involved than the anterior teeth; usually, all teeth distal to the most mesial affected tooth get affected; deciduous and permanent molars are likely to be involved and usually present unilaterally.
- Gender predilection of Male: Female of 1: 2.25
- Radiographic sign: Resorption chimneys, which are enlarged bony crypts around the tooth‐germ crowns formed due to resorption of the alveolar process are seen. Ankylosis may be a secondary feature.

**FIGURE 1:**
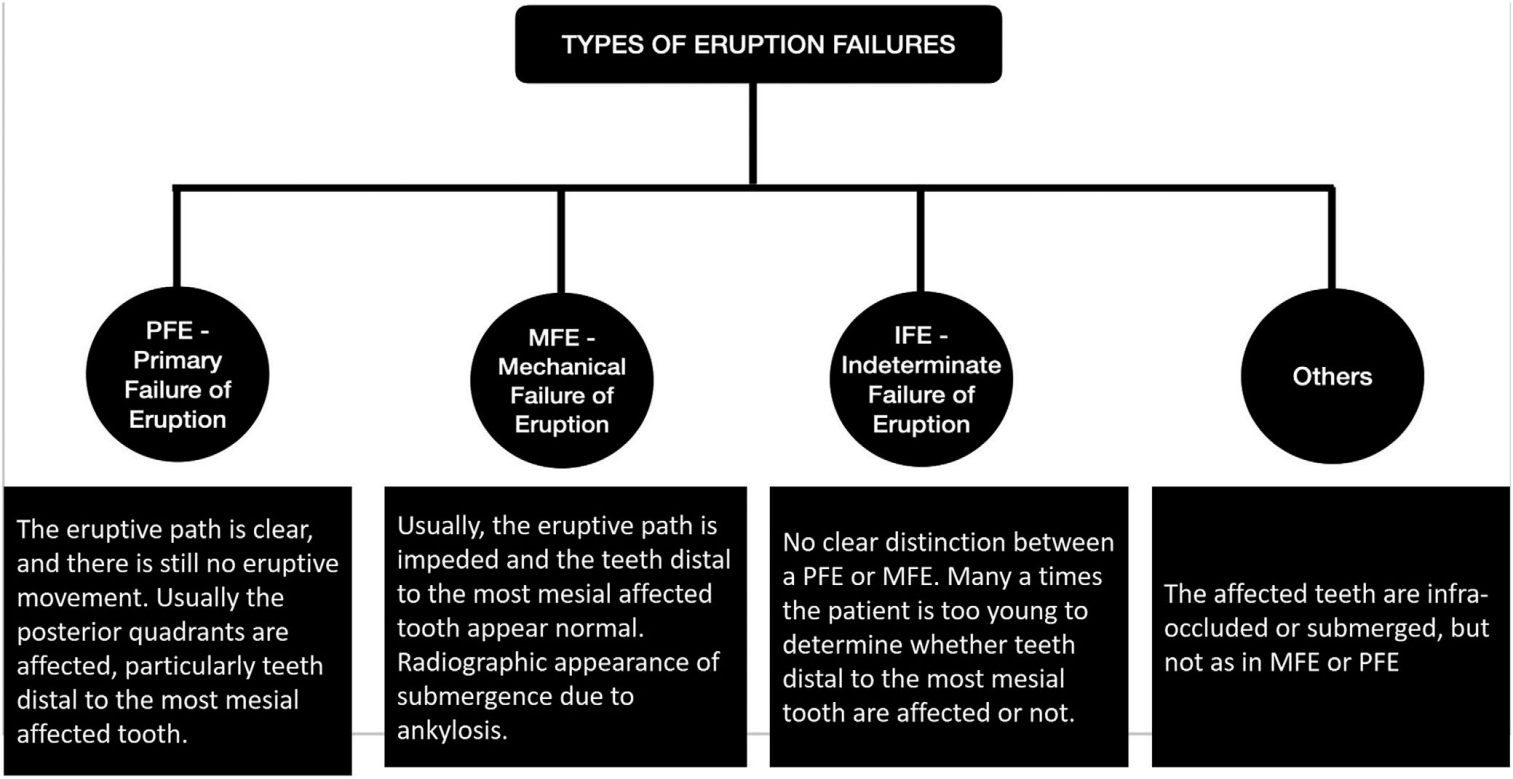
Types of eruption failures

Frazier‐Bowers et al.^13^ have categorized the diagnostic characteristics of posterior open bite based on different types of eruption failures (Figure 1). There appears to be a subtle difference between the different types, and definitive diagnosis is usually possible with other aids such as genetic linkage analysis. Though PFE appears as a complete failure of eruption without a distinct local or systemic etiology, mutations in parathyroid hormone receptor 1 (*PTH1R*) have been identified in several familial cases.

A more detailed clinical assessment identified two kinds of non‐syndromic PFE: type I and type II, both of which affect the posterior regions unilaterally or bilaterally. Type II is distinguished from type I by the greater eruption potential of the most distal tooth impacted by PFE.^9^ It is unclear if certain phenotypes are related to specific genetic variants or represent the broad range associated with *PTH1R*; however, it is well established that PFE does not react to orthodontic treatment, regardless of clinical severity or type.^9^, ^10^, ^11^

### 1.2 Pathogenesis of primary failure of eruption

Regulatory events of tooth eruption are confined to the dental follicle region that envelop the unerupted tooth. Molecular events during the active eruptive phase of tooth movement can be broadly approached as genetic and proteomic events. These control the critical pathways of osteoclastogenesis and osteogenesis, which are imperative for eruption. Despite the huge clinical interest behind PFE, the core pathogenic mechanisms are yet to be deciphered. Most of the research pertain to assessment of specific genes involved. Many studies indicate mutation in the *PTH1R* gene, and about 51 mutations of the *PTH1R* gene have been reported till date.^9^, ^12^, ^13^, ^14^, ^15^, ^16^, ^17^, ^18^

Several genes have been identified that play a role in odontogenesis including PAX9, MSX1, *PTH1R*, and AXIN2. Strong evidence exists that, in most of the cases, PFE is an autosomal dominant heterogeneous condition associated with mutations in *PTH1R* gene and the genes involved in activation of cAMP/PKA pathway in tooth eruption. However, not all patients with PFE carry mutations in known genes and the underlying genetics of PFE is still unexplored.^19^, ^20^

Transitory alteration to RANKL functions during the initial stage of dental root elongation and tooth eruption have also been reported in mouse models of PFE.^21^In‐depth analysis of chronological regulation and spatial localization of genes is key for improved understanding. Also, validation of the protein expression is essential for derivation of therapeutic interventions.

Other risk factors that have been consistently reported are a strong family history of PFE in 10%–40% of PFE. The presence of virus in nerve tissues in the perifollicular region has also been studied but disregarded due to lack of evidence.^9^

### 1.3 Treatment options

Treatment of patients with eruption failures can often be challenging. An interdisciplinary approach is required when considering the management of this rare condition. The various treatment options include the following^22^, ^23^, ^24^, ^25^, ^26^:

1. Accept the infraocclusion.
2. Restorative correction of the occlusion once growth has ceased.
  - Coronal build‐up or onlay of the affected teeth.
  - A removable prosthesis over the affected teeth.
3. Extraction of affected teeth and prosthetic replacement.
4. Surgical repositioning of the affected area with a segmental osteotomy once growth has ceased, although limited success has been reported using this approach

Exposing and bonding teeth affected by PFE are not recommended as treatment with orthodontic forces has been suggested to lead to localized ankylosis.

## 2 CASE HISTORY AND DIAGNOSIS

**FIGURE 2:**
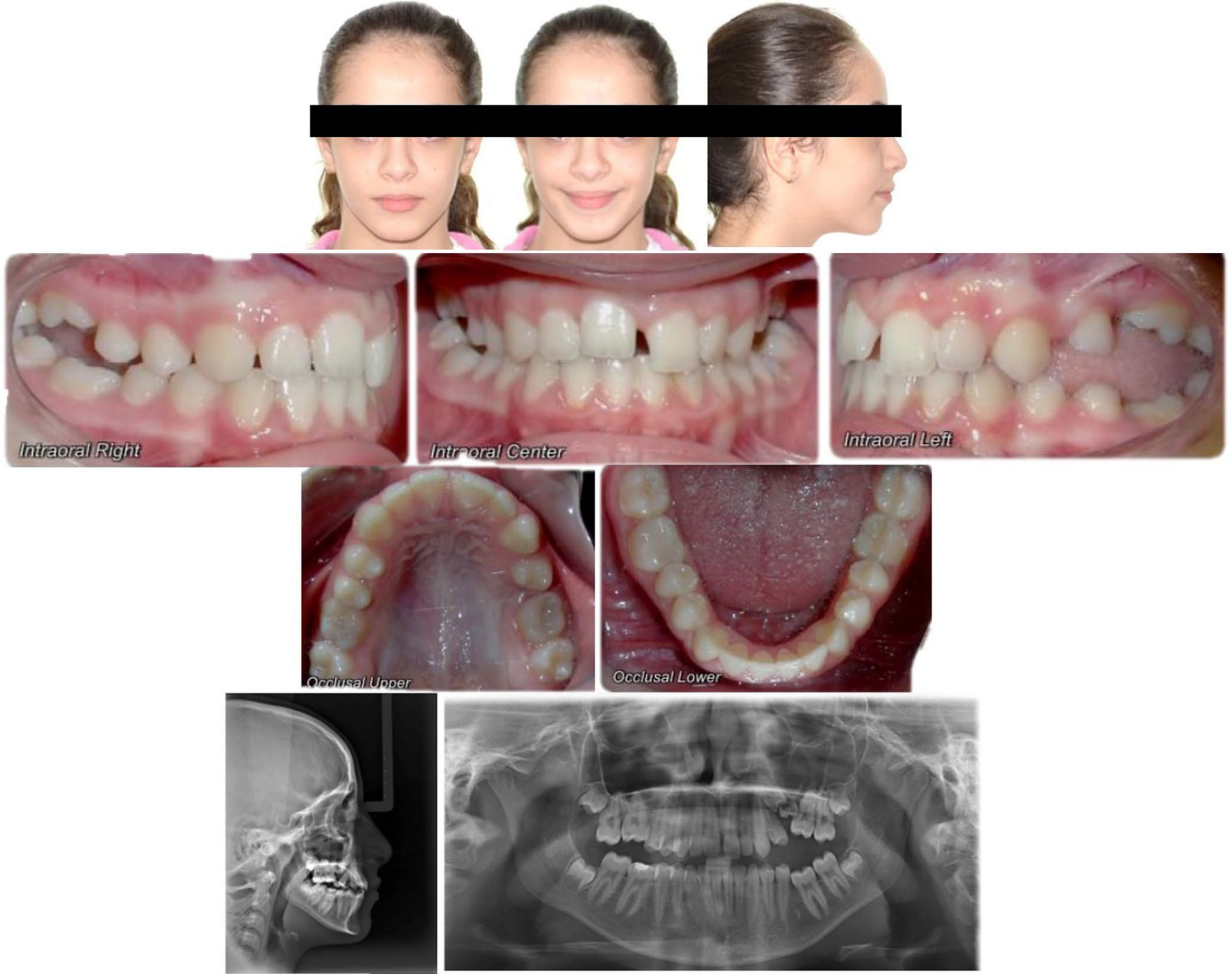
Initial treatment records with radiographs

A 13‐year‐old girl reported to us with multiple spaces between her front teeth and complained of difficulty in chewing. Clinical examination revealed a lack of lip fullness, bilateral posterior open bite with multiple submerged and infra‐occluded posterior teeth. All the permanent teeth except for the upper left second premolar were seen intraorally. Orthopantomogram (OPG) X‐ray revealed infra‐occluded first molars on all quadrants without any mechanical obstruction except for the second quadrant where in it seemed that an unerupted retained second deciduous molar was obstructing the eruption of an impacted second premolar and infra‐occluded first molar. None of the infra‐occluded or impacted teeth showed any radiological signs of ankylosis (Figure 2).

**FIGURE 3:**
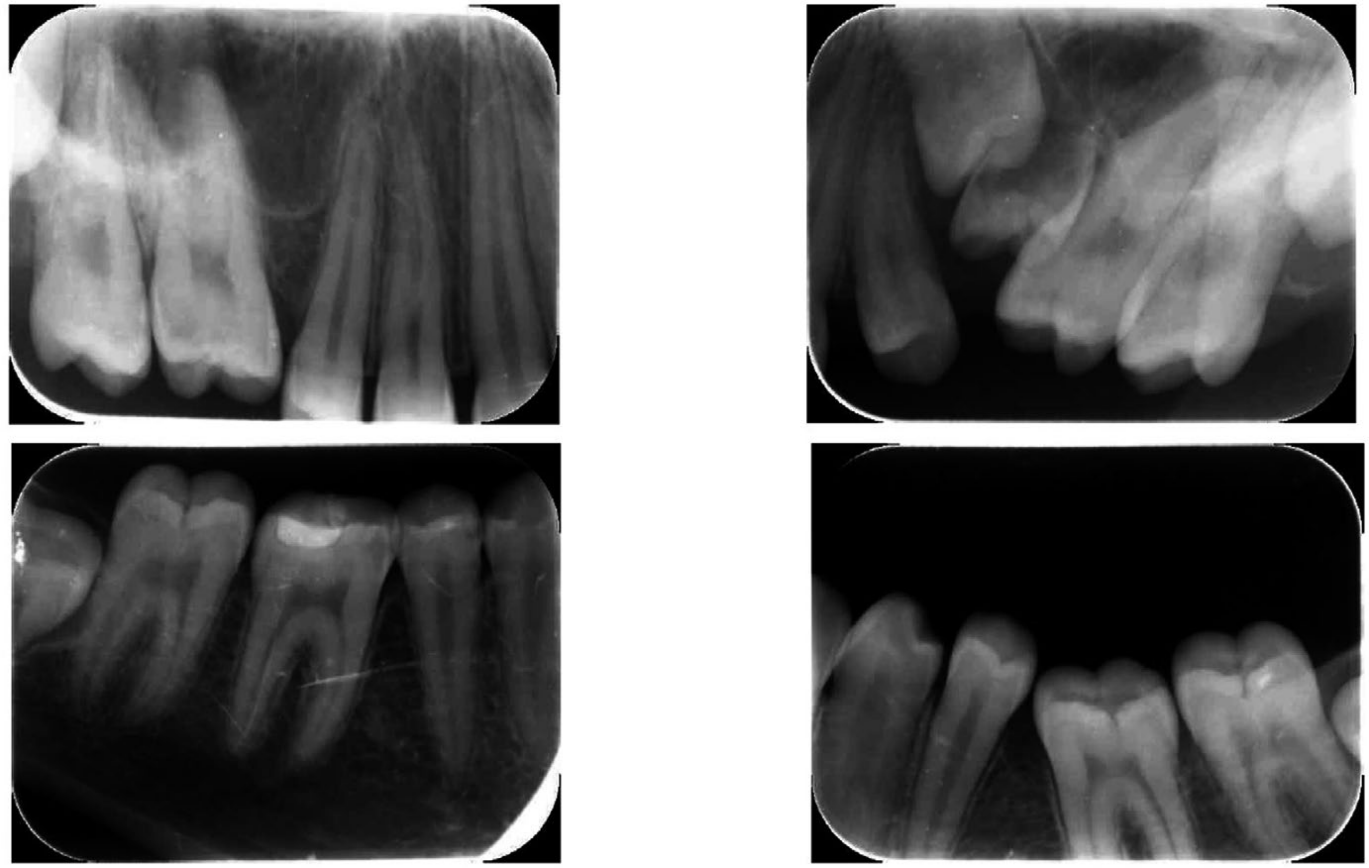
Periapical radiograph confirming normal periodontal ligament space and complete root formation of all her infra‐occluded and impacted teeth

There was no significant underlying medical history. No one in her family had a history of unerupted teeth. She demonstrated a Class I malocclusion with a convex profile. The patient reported no significant or unusual variations in the eruption pattern and the eruption timings of her teeth on both sides (Table 1). A further periapical radiograph confirmed normal periodontal ligament space and complete root formation of all her infra‐occluded and impacted teeth. No relevant bone pathology was evident either (Figure 3). Correlating the above clinical findings and radiological findings, a provisional diagnosis of eruption failure was made. Since there was no history or any other syndromic or familial involvement, a therapeutic diagnostic approach was followed during the treatment.

**TABLE 1:** Cephalometric analysis

Variable	Pre‐treatment	Post‐treatment
FMA (dg)	28.64	28.33
SNA (dg)	80.4	81.5
SNB (dg)	76.9	79.3
ANB (dg)	3.5	2.2
IMPA (dg)	86.16	77.5
U1‐SN (dg)	98.32	106.18
NLA (dg)	95.35	95.98
Interincisal angle (dg)	132.76	134.54
Rickett's E line (mm)	0.26	−1.00

## 3 TREATMENT PLAN AND PROGRESS

Due to the presence of multiple spaces in the anterior region and a mechanical obstruction in the form of an unerupted retained second deciduous molar on the second quadrant, it was decided to use a fixed orthodontic appliance to close the anterior spaces and get the impacted #25 into occlusion without involving the first molars in the continuous arch.

A *wait and watch* approach was implemented wherein initially all the molars would be aligned without creating any extra space. If the molars would be unresponsive to the initial orthodontic forces, extraction of all first molars would be performed following which the left upper deciduous second molar would be extracted, and all the spaces would be closed by protracting the second molars. At this point, it is assumed that extraction of the left upper deciduous second molar should be able to clear the path for eruption for the second premolar.

**FIGURE 4:**
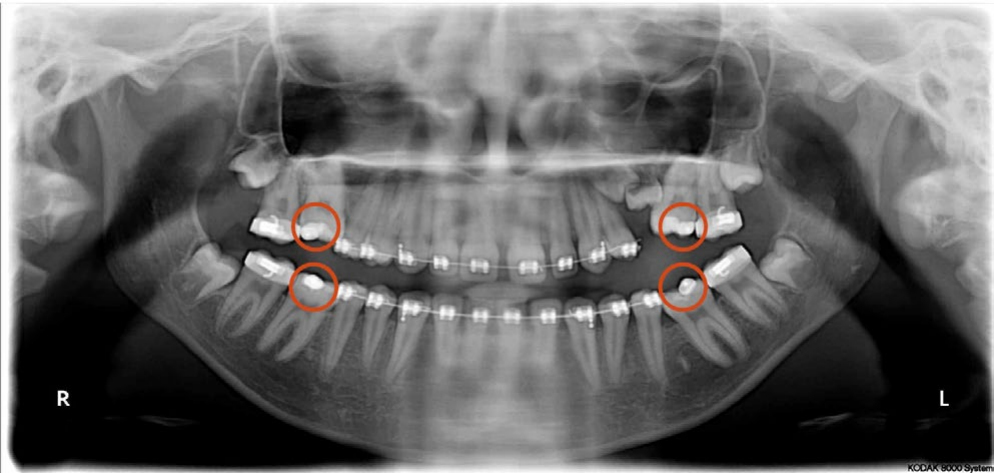
Orthopantomogram showing four lingual buttons on the infra‐occluded first molars for inter‐arch traction using intermaxillary elastics

This case was treated using 0.022′′ × 0.028″ slot pre‐adjusted edgewise appliances with MBT prescription. Initial leveling was performed to align the arches and get the molars to the occlusal level. Initially, the molars were not included in the main arch with the fear of further increasing the posterior open bite. Lingual buttons were placed on the buccal surfaces of the first molars, and intermaxillary elastics 1/8 2 Oz were used from the upper molars vertically to the lower molars in order to extrude them (Figure 4).

Unfortunately, even after six months of leveling using a sequence of NiTi archwires, no change in the position of the molars was noticed.

It was then decided to extract all the first molars and the deciduous second molar on the upper left quadrant (for unhindered eruption of the #25) and to protract the second molars into the extraction spaces.

**FIGURE 5:**
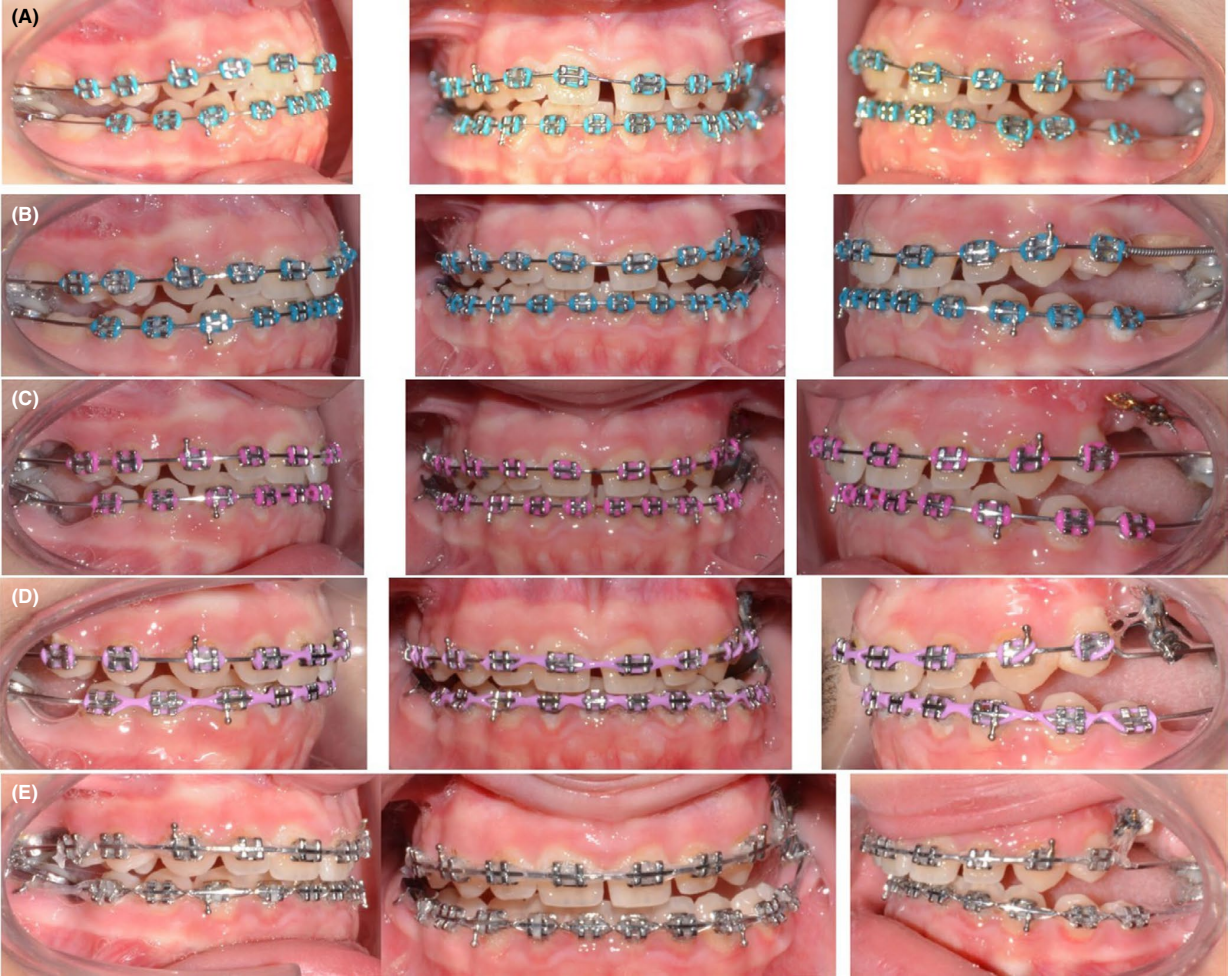
Treatment progress pictures. (A,B) Before extraction of first molars; (C‐E) After extraction of first molars

After waiting for a couple of months, when the #25 showed no signs of eruption, it was decided to extrude it orthodontically. A lingual button was placed on the exposed surface of the #25, and a power chain was tied from the button onto a stiff upper base arch wire (0.019′′ × 0.025′′ SS) in order to provide vertical traction (Figure 5C‐E).

**FIGURE 6:**
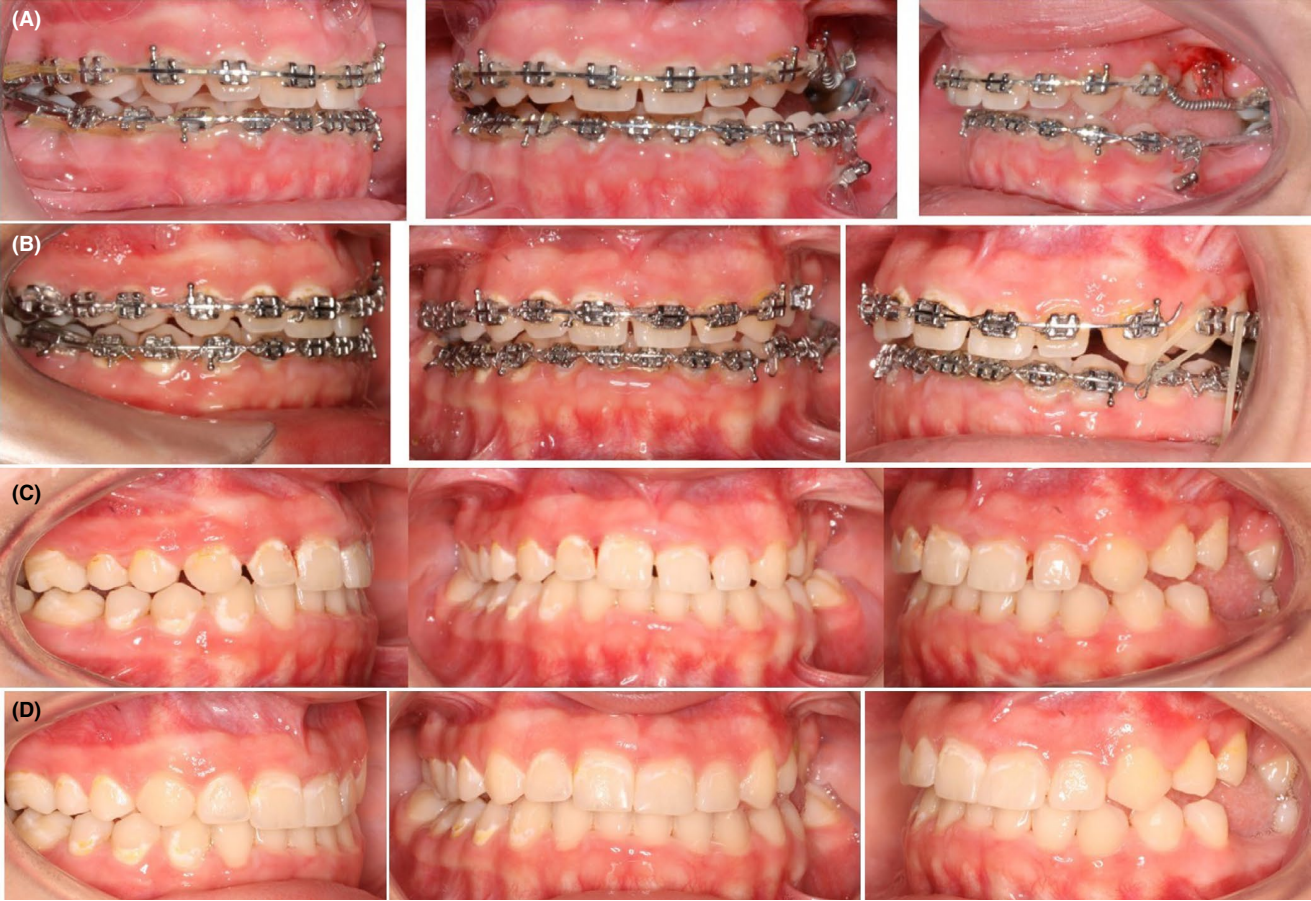
(A,B) Interarch traction to extrude the upper left second premolar. (C) Intraoral pictures at deband. (D) Intraoral pictures after composite restorations

The impacted #25 responded to the orthodontic force and began moving occlusally. The second molar on the right side was responding well to orthodontic protraction but the second molar on the left side was not. After 12 months of active treatment, both upper and lower second molars on the right side were successfully protracted, but the spaces on the left remained the same (Figure 6A,B).

Once the upper left second premolar erupted out of the oral mucosa, a TAD (1.6 × 8 mm) was placed on the opposing arch to facilitate further traction using inter‐arch elastics.^27^, ^28^ Although traction was initiated for a couple of months, it was noticed that the #25 became unresponsive to orthodontic forces (Figure 6A,B). Furthermore, even the right side became unresponsive to any kind of orthodontic force. In addition, any further diastema closure and occlusal settling were not possible either. Eventually, after achieving what could best be done using fixed orthodontic treatment for the patient, it was decided to deband the appliance. The remaining spaces were closed using composite restorations to provide better esthetics (Figure 6C,D).

## 4 TREATMENT RESULTS

**FIGURE 7:**
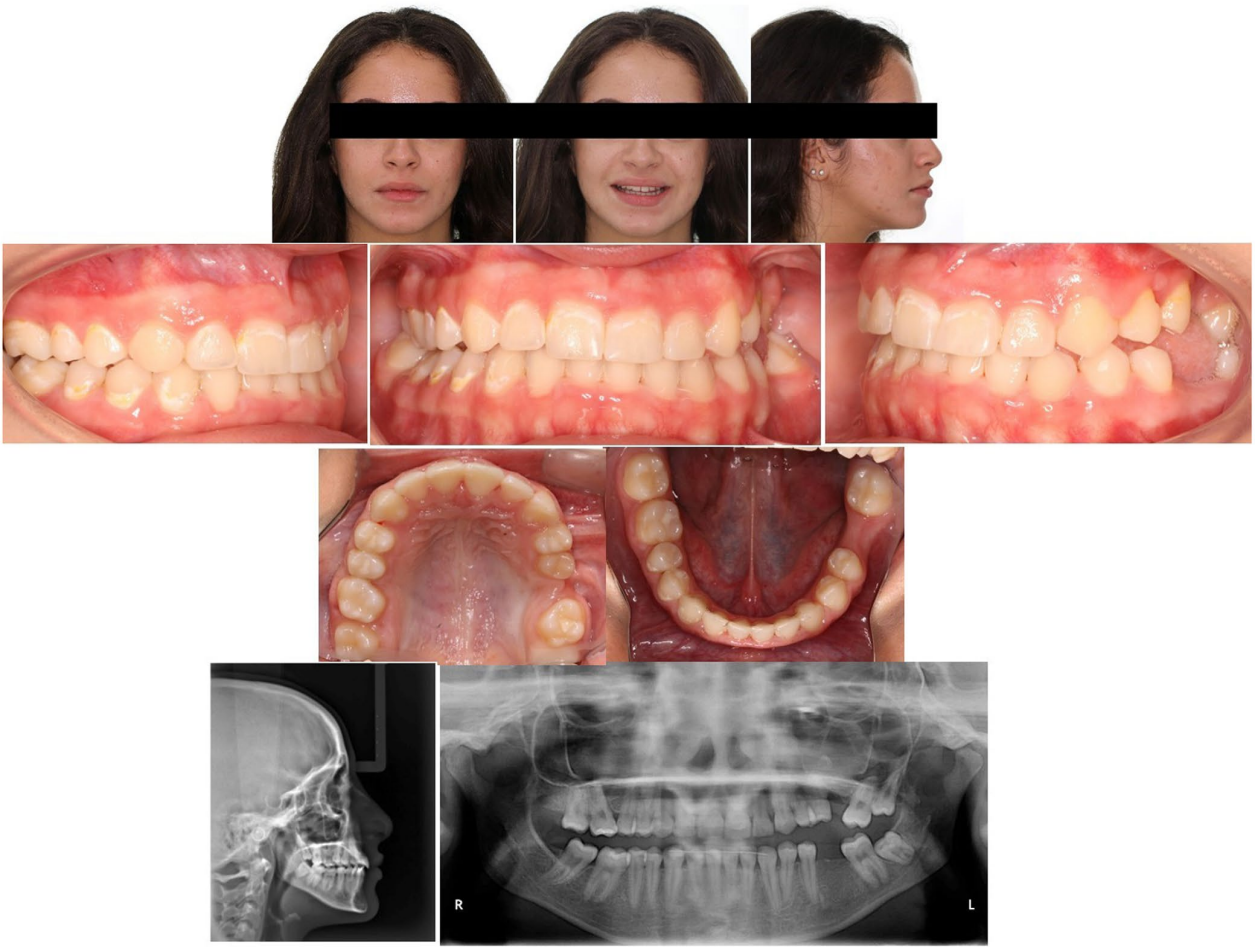
Post‐treatment extra‐ and intra‐oral pictures with radiographs

**FIGURE 8:**
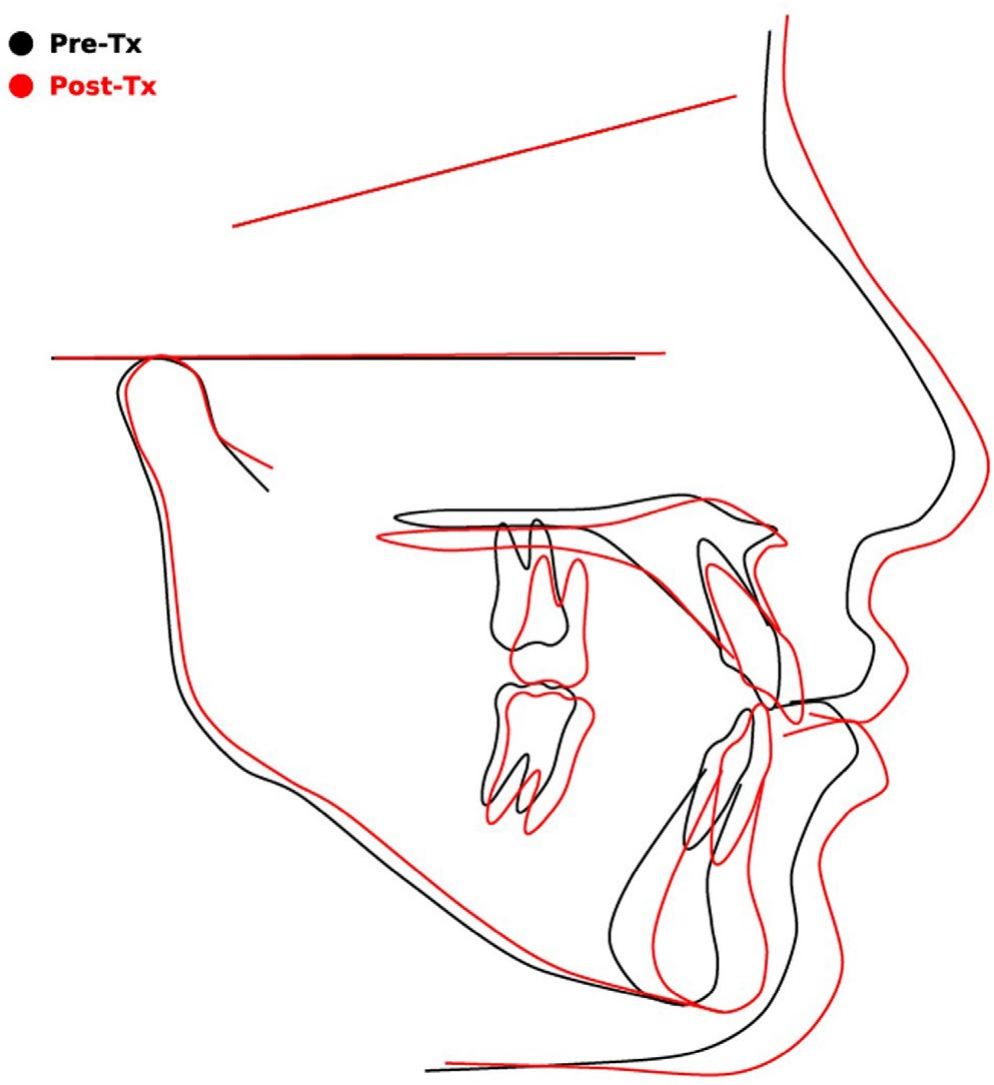
Superimpositions of the pre‐ and post‐treatment cephalograms

Post‐treatment intra‐oral photographs showed good alignment, a reasonable relationship between upper and lower teeth, and ideal overbite. A much more aesthetic and pleasing smile was achieved along with harmony between the upper and lower lips, lip competence, coincident dental midlines, and no muscle or joint problems during the treatment. All the spaces on the right side were successfully closed, but on the left side, most of the spaces remained on the upper and lower arch (Figure 7). Post‐treatment cephalometric analysis showed that the sagittal jaw relationship improved significantly, and the improvement in maxillo‐mandibular relationship can be partly attributed to the completion of growth (pre‐SNA, 80.4; post‐SNA, 81.5; pre‐SNB, 76.9; post SNB, 79.3; pre‐ANB, 3.5; post‐ANB, 2.2) while the mandibular plane remained almost constant (pre‐FMA, 28.64; post‐FMA, 28.33). There was a reduction in the lower incisor inclination (pre‐IMPA, 86.16; post‐IMPA, 77.5) and an increase in the upper incisor inclination (pre‐U1‐SN, 98.32; post‐ U1‐SN, 106.18). The increase in the U1‐SN signifies improvement and correction of the incisor inclinations to near normal that was achieved through labial tipping and third order corrections of the upper anterior teeth. The overjet was increased (pre, 2; post, 3.5), and overbite was corrected (pre, −0.6; post, 1.3; Table 1; Figure 8).

The patient was provided with a fixed lingual retainer along with an Essix retainer on the upper and lower arch to maintain the corrected teeth positions. Routine check‐ups were emphasized to closely monitor the treatment results.

## 5 DISCUSSION

### 5.1 Diagnostic challenges

Diagnosis of PFE poses a significant challenge due to the high degree of variability involved with its clinical presentations. Familial and isolated cases also show a good amount of variations symptomatically. First, an obstruction must be ruled out.^29^ Following this, factors like an underlying supernumerary tooth, retained deciduous tooth, cystic lesions, and bone calcifications must be ruled out. It may also be necessary to check for certain habits like a lateral tongue thrust or digit sucking that may predispose the formation of a posterior open bite by inhibiting the natural eruption of the posteriors.^1^, ^3^

It may be imperative to rule out the possibility of a syndrome associated with such submerged/ unerupted teeth before proceeding with the treatment. Some syndromes associated are listed in Table 2.^30^

**TABLE 2:** Syndromes associated with failure of eruption^30^

Cleidocranial dysplasia (OMIM 119600) Osteopetrosis (OMIM 166600/259700) Rutherford syndrome (OMIM 180900) GAPO syndrome (OMIM 230740) Osteoglophonic dysplasia (OMIM 166250) Dental non‐eruption (OMIM 125350)

In the absence of any such abovementioned etiological factors, a primary failure of eruption (PFE) may be a definitive diagnosis. To differentiate between the PFE and MFE conditions, involvement of the distal most teeth should be considered. In the presented case, the distal most tooth was erupted and reached the occlusal table and hence was pointing toward PFE.^9^, ^31^

A classic diagnostic feature proposed by Rhoads et al.^32^ is the frequent involvement of the first molar along with the adjacent teeth in an unusual supracrestal position. A recent systematic review by Hanisch et al.^9^ identified bilateral infraocclusion of the posterior teeth as a hallmark feature of eruption failure. Although genetic testing and identification of mutation in *PTH1R* gene would confirm the diagnosis of PFE, absence of mutation would not necessarily rule out PFE.

According to the diagnostic rubrics,^12^, ^33^ our patient had the following radiological/clinical signs:

1. The teeth of the posterior region are affected more frequently (first and second molars more often than premolars or canines)
2. Affected teeth were resorbing the alveolar bone coronal but do not erupt totally or erupt incomplete
3. Mostly the clinical findings were asymmetric, which means there was a bilaterally unbalanced eruption of the teeth.
4. Vertically, there is was an impairment of growth of the alveolar bone in the affected region
5. A severe lateral open bite in the affected region

The probability of this patient having a *PTH1R* variant is greater as five specific clinical characteristics were present.

### 5.2 Treatment strategies

Evidence suggests that any tooth affected with PFE will not respond to orthodontic forces. If the lateral open bite is to be corrected with orthodontic forces, then it will lead to ankylosis of the affected teeth. This may act as an anchor, and an extrusive force on the anchor may cause intrusion of adjacent teeth. Usually, such attempts will worsen the existing open bite. To prevent such iatrogenics, it was decided not to involve the infra‐occluded first molar in the continuous mechanics.^3^, ^7^, ^34^ When a patient with PFE undergoes orthodontic treatment, it may be difficult to keep them motivated. In such cases, it is critical to maintain modest treatment goals rather than attempting to reach perfection. Although the cosmetic outcome may not be similar to that of a normal patient, depending on the severity of the condition, additional treatment options such prosthodontic or restorative options may be considered.

Treatment choice for PFE must be considered after evaluating the patient's age and the severity of PFE. For growing children, a conclusive treatment may not be possible until the vertical growth is completed. However, a regular observation is necessary. In the case treated above, since the patient reported to us during growing stage, it was planned to close the other spaces and prepare the patient for a permanent prosthesis. In such growing patients, direct or indirect composite buildups could also ensure occlusal stability and preserve alveolar bone level until an implant placement is possible.^35^

In adult patients with only mild infraocclusion, it is prudent to accept the occlusion as it is and attempt no treatment. When affected teeth are partially erupted in the oral cavity, overlay crowns or overlay dentures can be considered as treatment options. In some cases, removal of the affected tooth may be the treatment of choice.^36^ Yasumura and Sueishi^37^ reported a case of maxillary first molar that failed to erupt after fenestration and responded negatively to orthodontic forces. However, extraction of the affected first molar resulted in mesial migration and spontaneous eruption of the unerupted second molar. Further orthodontic treatment resulted in functional occlusion. A similar approach was followed in the above patient wherein we tried attempting protraction of second molars after extraction of first molars. It was successful on right side and failed on the left side.

Single tooth osteotomies have been promising as an alternate approach to correct the open bite related to PFE. This along with immediate elastic traction will utilize the regional acceleratory phenomenon. Shirota et al.^38^ reported a case of 24‐year‐old male patient with unilateral posterior open bite secondary to PFE of maxillary premolars and molars. The patient was treated successfully with segmental osteotomy along with alveolar distraction. An alternative orthodontic treatment approach is to employ a segmented mechanics with pre‐bonding prophylaxis and adequate bracket detachment measures,^39^, ^40^ and avoid continuous arch wire and leaving the infraocclusion and related open bite in the molar region uncorrected.

## 6 CONCLUSION

When faced with failure of eruption, it is important to have a hawk's eye while diagnosing and treating the patient. A careful observation of the presentation and the responses of teeth during treatment are very vital. We must be mindful of the true etiology, given the similar clinical presentations of PFE, MFE, and impactions. Impacted teeth should erupt once the physical obstruction is removed; however, teeth affected by PFE and MFE will not. It is important to remember that applying orthodontic traction to teeth affected by PFE will not be successful and, indeed, may cause ankylosis. A multidisciplinary approach is required to treat such conditions. This correspondence highlights the relevant literature around eruption failures and demonstrates the treatment of a case of PFE that was treated to the best possible outcome.

## CONFLICT OF INTEREST

The authors declare no conflict of interest.

## AUTHOR CONTRIBUTIONS

Mirna G Awad and Lana Dalbah treated the case. M. Srirengalakshmi was involved in writing the case report. Adith Venugopal and Nikhilesh Vaid were involved in supervising, diagnosing, treatment planning of the case, and proofreading the manuscript.

## ETHICAL APPROVAL

This study did not require an ethical approval from any review board.

## CONSENT

The signed consent form is available with the principal author.

## Data Availability

The data used to support the findings of this study are included within the article.
